# Exopolysaccharide-Producing *Bifidobacterium adolescentis* Strains with Similar Adhesion Property Induce Differential Regulation of Inflammatory Immune Response in Treg/Th17 Axis of DSS-Colitis Mice

**DOI:** 10.3390/nu11040782

**Published:** 2019-04-04

**Authors:** Rui Yu, Fanglei Zuo, Huiqin Ma, Shangwu Chen

**Affiliations:** 1 Key Laboratory of Functional Dairy Science, College of Food Science and Nutritional Engineering, China Agricultural University, Beijing 100083, China; hardheartyr@sina.com (R.Y.); duruolanzi@gmail.com (F.Z.); 2 College of Horticulture, China Agricultural University, Beijing 100193, China; hqma@cau.edu.cn; 3 The Research & Innovation Centre of Food Nutrition and Human Health (Beijing), China Agricultural University, Beijing 100083, China

**Keywords:** *Bifidobacterium adolescentis*, macrophage, Treg/Th17 axis, exopolysaccharide, ERK/p38 MAPK pathway, IBD

## Abstract

Intestinal bifidobacteria benefit human health by promoting and modulating the gut flora, and boosting therapeutic efficiency for chronic metabolic diseases and cancer. Recently, *Bifidobacterium adolescentis* strains with high adhesion to intestinal epithelial cells were associated with induction of T-helper 17 (Th17) cells in humans and rodents. Here, two *B. adolescentis* strains with similar adhesive ability but different aggregation properties were investigated for specific immunoregulatory effects, including the underlying cellular pathway, on macrophage and T-regulatory (Treg)/Th17 axis activation in vitro and in the colon of dextran sodium sulfate (DSS)-colitis mice in vivo. In-vitro, the auto-aggregative *B*. *adolescentis* strain IF1-11 induced significantly higher IL-6 and lower IL-10 secretion from immune cells, and it induced abundant Th17 cells. The non-aggregating strain IF1-03 induced significantly higher IL-10, less IL-6 and a high proportion of Treg/Th17 cells compared to total T cells. In vivo, orally administered IF1-03 protected DSS-colitis mice via activation of dendritic cells or macrophages and skewing of Treg/Th17 cells, consistent with Treg cell induction in vitro. IF1-03 exopolysaccharides showed a functional recognition pattern similar to IF1-03 for IL-10 cytokine secretion and Treg cell-differentiation induction, both dependent on the toll-like receptor 2–ERK/p38 MAPK-signaling cascade for macrophage activation. We suggest that *B*. *adolescentis* exopolysaccharide-associated enterocyte adhesion/aggregation phenotypes determine strain-specific adaptive immune responses in the gut via the macrophage-regulated Treg/Th17 axis.

## 1. Introduction

Microorganisms colonize all surfaces of the human mucosa. Numbering one hundred trillion [1] and representing the second genome of the human body, the genetic potential of gastrointestinal microbiota outnumbers that of the host by two orders of magnitude [2]. Studies have proven that gastrointestinal microbiota have beneficial integral functions in many chronic metabolic diseases in humans, including inflammatory bowel disease (IBD), diabetes [3], obesity, cancer, hypertension [4], and chronic kidney [5] and cardiovascular [6] diseases. One of the first colonizers of the human gut, *Bifidobacterium* has been well studied for its effect on the endogenous microbiota through modulation of cell metabolism, epithelial barrier function and short-chain fatty acid metabolites such as acetate [7], as well as for its critical role in controlling the cancer response to immunotherapy [8].

In the last two decades, the indirect and direct regulatory effects of probiotic strains on the immune response of innate and adaptive immune cells have been evaluated [9]. Innate immune cells, including macrophages and dendritic cells (DCs), detect microorganisms and respond to pathogen- and microorganism-associated molecular patterns (PAMPs and MAMPs, respectively) when the bacteria are translocated across the intestinal mucosa [8,10]. The activated macrophages and DCs produce nitric oxide (NO) and other reactive oxygen intermediates, secrete cytokines, and present antigens to direct T-cell proliferation and differentiation and induce adaptive immune responses. Gut microbiota have been reported to shape the T regulatory/T-helper 17 (Treg/ Th17) axis of adaptive immune cells, which functions to protect the host from pathogenic microorganisms and viruses and restrain an excessive effector T-cell response in intestinal mucosa, thus restoring, for example, intestinal homeostasis in IBD patients [11]. Germ-free and antibiotic-treated mice have defects in the development of their immune system and manifest a paucity of intestinal Treg and Th17 cells. On the other hand, reports have documented an increase in colonic Treg or Th17 cells after inoculating with fecal material from healthy individuals or patients with colitis [12,13]. For instance, *Bifidobacterium pseudocatenulatum* CECT7765 administration to obese mice fed a high-fat diet reduced systemic inflammation by restoring the balance of Tregs and B lymphocytes and reducing the proinflammatory cytokines interleukin 17A (IL-17A) and tumor necrosis factor alpha (TNF-α) [14]. Although studies have revealed the importance of microbial signals for the maintenance of microbiota-dependent immune homeostasis, and it is generally accepted that the immunoregulatory effect is strain-specific, investigation of the precise mechanisms through which the microbes exert their influence is only in its infancy [15]. Adhesion ability to intestinal epithelial cells has been a critical criterion for selection of probiotics from *Lactobacillus* and *Bifidobacterium* strains [16]. A recent study revealed an association between Th17 cell induction and adhesion to intestinal epithelial cells of commensal microbe strains, such as segmented filamentous bacteria and 20 bacterial strains isolated from patients with ulcerative colitis [12]. *Bifidobacterium adolescentis* was the first identified human symbiont bacterial species that could induce Th17 cells in murine intestine and was closely associated with the gut epithelium [17]. Taken together, there is renewed interest in bacterial species’ physicochemical properties, such as adhesion ability, as related to the Treg/Th17 axis.

Although having distinct effector functions, Treg and Th17 cell lineages share similar cytokine requirements for their differentiation from naïve CD4^+^ T cells and they are reciprocally regulated by key mediators, such as transforming growth factor beta (TGF-β), IL-6 and IL-10, which are secreted by innate immune cells [18,19]. TGF-β induces transcriptional upregulation of both *Foxp3* and *RORγt*, transcription factors that direct the Treg and Th17 cell-differentiation programs, respectively, whereas in the presence of proinflammatory cytokines (IL-6, IL-21, or IL-23), *RORγt* expression is further upregulated and *Foxp3* is inhibited [20]. IL-10 is responsible for maintaining the expression and function of Foxp3 in Treg cells [21]. Therefore, the cytokine pattern of IL-6, IL-10 and TGF-β induction by microorganisms is critical for Treg/Th17 cell balance [22]. *Bifidobacterium bifidum* strains showed Th17-profile cytokines in the peripheral blood mononuclear cells, while these strains induced the differentiation of Treg cells from naïve lymphocytes in strain-stimulated DCs [23]. Therefore, the in-vitro cytokine profile of the immune system is important in investigating the immunoregulatory response of microorganisms.

Microbial activation of innate immune cells involves recognition of P/MAMPs by the pattern-recognition receptors (PRRs) of macrophages, DCs and epithelial cells, transitioning the signal to induce the innate and adaptive immune response downstream, and pro/anti-inflammatory cytokine induction [24,25]. Toll-like receptors (TLRs) are a multimember family that is mainly involved in pattern recognition reception. In mammals, the TLR2/TLR1 or TLR6 heterodimer coordinates macrophages and DCs to recognize P/MAMPs, such as Gram-positive bacterial sugar-complexed PAMPs [26,27,28,29]. Along with the signal-transduction networks, nucleosome remodeling and covalent histone modifications are implicated in regulation of the targeted gene, both in induction and transcription. The mammalian *Il10* gene promoter contains complex enhancer *cis*-elements of different transcriptional factors. Histone acetylation is associated with transcription activator accessibility to chromatin binding. Macrophage activation by bifidobacterial-produced components, and specifically *B. adolescentis* strain-dependent immune cell activation, and the different signals emerging for cytokine induction and CD4^+^ cell differentiation have never been explicitly illustrated.

In our previous study, we characterized the general probiotic properties, such as antibiotic resistance, and adhesion and aggregation ability, of 13 *Bifidobacterium* strains isolated from breast-fed infant feces, and identified four *B. adolescentis* strains with different bacterial phenotypes in terms of auto-aggregation, bacterial pathogen coaggregation ability and intestinal epithelial cell adhesive capacity, in in-vitro assays [30]. In this study, we investigated these four *B. adolescentis* strains’ immunoregulatory effect on the adaptive immune response, compared their potent probiotic abilities and elucidated the underlying cellular and molecular mechanisms involved. We looked at the *B. adolescentis* strains’ stimulation of NO secretion and cytokine profiles in macrophages, and their immunoregulatory effects on Treg and Th17 cell induction and expansion, and specifically for mice with IBD, their strain-dependent protection of the Treg/Th17 axis of the cellular immune response system, in vitro and in vivo. The underlying mechanisms of strain-specific exopolysaccharides (EPSs), macrophage TLR2 activation, and extracellular signal-regulated kinase (ERK)/p38 mitogen-activated protein kinase (MAPK)/nuclear factor kappa B (NF-κB) signaling cascades for Treg/Th17 cell expansion, induction and function were deciphered.

## 2. Materials and Methods

### 2.1. Bifidobacterial Strains, Cell Lines and Animals

#### 2.1.1. Bifidobacterial Probiotic Strains

The bifidobacterial strains used in this study were isolated and purified from feces samples of breast-fed infants and maintained in our laboratory (Table 1) [30]. *Bifidobacterium animalis* subsp. *lactis* BB-12 was obtained from Chr. Hansen (Beijing) Trading Co., Ltd. (Beijing, China).

#### 2.1.2. Macrophage Cell Line

Cells from the murine macrophage cell line RAW264.7 (ATCC SC6003) were obtained from the American Type Culture Collection (ATCC, Rockville, MD, USA), and routinely cultured in DMEM (Gibco, Grand Island, NJ, USA) supplemented with penicillin–streptomycin (100 U/mL, Gibco, Grand Island, NJ, USA) and 10% (*w*/*v*) heat-inactivated fetal calf serum (FCS; Gibco) at 2–4 × 10^4^ cell/cm^2^, 37 °C, in a humidified atmosphere (95% relative humidity) and 5% CO_2_. 

#### 2.1.3. Experimental Animals

Specific-pathogen-free (SPF) male BALB/c mice aged 6–8 weeks, and 10- to 12-week-old SPF C57BL/6 mice were obtained from Weitong Lihua Experimental Animal Technology Co. Ltd. (Beijing, China) (Certification of Experimental Animal, CAE no.: SCXK (Jing) 2016-0001), and maintained with free access to food and water at 20 ± 2 °C, relative humidity of 55%, under a 12 h light–dark cycle. Animal care and experimental procedures were carried out in accordance with the Use and Care of Laboratory Animals (People’s Republic of China) and supervised by the Agricultural/Institutional Animal Care and Use Committee (IACUC) of China Agricultural University (CAE no.: SCXK (Jing) 2018-5001-4).

### 2.2. Bifidobacterial Strains and Immunocyte Cell Preparation and Culture

#### 2.2.1. Probiotic Strain Culture

All probiotic strains—*B. adolescentis* IF1-11 and IF1-03, and *B. animalis* subsp. *lactis* Bb12 (Table 1)—were grown overnight in MRS broth (Difco Laboratories, Detroit, MI) supplemented with 0.05% (*w*/*v*) L-cysteine under anaerobic conditions (10% H_2_, 10% CO_2_, 80% N_2_). Cultures were harvested by centrifugation at 6000× *g*, washed three times with sterile phosphate buffered saline (PBS, pH 7.4), and final bacterial dilution was according to bacterial colony forming units (cfu/mL) at the indicated bacterial cell densities in different buffers or media. Bacteria suspended in DMEM (Dulbecco’s Modified Eagle Media) were used to stimulate RAW264.7 macrophage cells and splenocytes in in vitro coculture assays. For oral administration to mice, bacteria were resuspended in skim milk at 5 × 10^9^ cfu/mL. Bacterial density (cfu/mL) was determined by standard plate counting as previously described [30], and the diluted density was determined by using a Thoma cell-counting chamber, in duplicate.

#### 2.2.2. Spleen Lymphocyte Preparation, T-Cell Stimulation and Flow Cytometry 

Splenocytes were collected from male BALB/c mouse (6–8 weeks old) spleens by standard techniques with PBS flushing. Splenocytes were gently scraped with a 1-mL syringe and passed through a 40-mm nylon cell strainer (BD Falcon, San Jose, CA, USA). Erythrocytes were removed using red blood cell lysis buffer (Biyuntian Biotech, Beijing, China). Splenic lymphocytes (2 × 10^6^ cells) were cultured in 6-well plates precoated with anti-mouse CD3 (10 μg/mL, clone 145–211) and anti-mouse CD28 (2 μg/mL, clone T2.5) antibodies (both from BioLegend, San Diego, CA, USA), and cocultured with or without bifidobacterial strains (1 × 10^7^ cfu) at a ratio of 5:1 splenocyte in RPMI-1640 medium (Gibco) supplemented with 10% FCS and 100 U/mL penicillin–streptomycin. Three days (72 h) later, cells were collected and stained with monoclonal antibodies CD4-PerCP-cy5.5 (clone GK1.5), CD4-FITC (clone GK1.5), Foxp3-PE (clone MF-14), CD25-APC (clone PC61.5) and IL-17-PE (clone TC11-18H10.1) (all from BioLegend). Fluorescent signals were acquired using a BD FACSCanto II flow cytometer (BD Biosciences, San Jose, CA, USA). Data were analyzed with FlowJo (Tree Star, Ashland, OR, USA) software.

### 2.3. Macrophage Treatments, and NO and Cytokine Assays

RAW264.7 macrophages were seeded in 24-well plates at 5 × 10^5^ cell/mL and incubated for 12 h. The washed and enumerated bifidobacterial strain cells (5 × 10^5^–10^7^ cfu/mL) were then inoculated into the plate to stimulate the macrophages at the specified ratios for 24 h. NO production in the medium was assessed by measuring nitrite/nitrate—the stable degradation products of NO—using Griess reagent (Biyuntian Biotech) according to the manufacturer’s protocol. The concentrations of TNF-α, IL-1β, IL-6, IL-10 and TGF-β in the supernatant were determined by a commercial ELISA kit (BioLegend) following the manufacturer’s instructions.

### 2.4. Dextran Sodium Sulfate (DSS)-Induced Colitis in Mice and Probiotic Protection

C57BL/6 mice (20 animals, 20 ± 2 g) were randomly assigned to four groups: one control, one DSS-colitis model and two DSS-bifidobacterial protection groups using strains IF1-11 and IF1-03, each with 5 animals per group. All mice received placebo (100 μL skim milk) or 5 × 10^8^ cfu live bifidobacterial strain cells (in 100 μL skim milk) daily, through an intragastric cannula, during the 15-day experimental period. DSS (3% *w*/*v*, 36–50 kDa, MP Biomedicals, Santa Ana, CA, USA) was given in the drinking water ad libitum from day 9–15 of the experiment to the DSS-colitis model and the two DSS-bifidobacterial protection groups. At the end of the experimental period (day 16), animals were anesthetized and killed by cervical dislocation.

Body weights of C57BL/6 mice were monitored and body weight increase was calculated according to the following formula: index (%) = (final body weight – initial body weight)/initial body weight. Other clinical signs of colitis, such as disease activity index, diarrhea, and bloody stool (fecal occult blood), were assessed and reported as a score from 0 to 4. Colon small segments or cross-sectional Swiss rolls were fixed in 10% formaldehyde and embedded in paraffin followed by hematoxylin and eosin staining. To analyze the cytokines secreted by the colonic lamina propria cells, colons were cut into short-length segments and washed twice in PBS containing 2 mM EDTA for 15 min at 37 °C under gentle stirring. After shaking twice in RPMI-1640 containing 1% (*v*/*v*) fetal bovine serum (FCS), 1 mM EGTA and 1.5 mM MgCl_2_ for 20 min at 37 °C, the lamina propria cells were digested in RPMI-1640 containing 20% FCS, 200 U/mL collagenase and 40 U/mL DNase I for 120 min at 37 °C under gentle stirring. Digested tissue was washed and filtered through a 40-mm nylon cell strainer (70 μm, BD Falcon) to obtain a single-cell suspension. Lamina propria cells were cultured in RPMI-1640 culture medium supplemented with penicillin (100 U/mL), streptomycin (100 U/mL) and 10% heat-inactivated FCS overnight at 37 °C and 5% CO_2_, and supernatants were collected for cytokine determination.

### 2.5. Immunofluorescence Microscopy 

Colon tissue sections (5–7 μm) were cut from paraffin-embedded colonic tissues, deparaffinized in dimethylbenzene and rehydrated with different concentrations of alcohol. Rehydrated sections were heated to 95 °C in 0.01 M citrate buffer for 15 min for antigen retrieval after permeabilization with 0.1% (*w*/*v*) NP-40 or 0.3% (*w*/*v*) Tween 20 in PBS for 10–15 min for Foxp3 or IL-10 and IL-17A staining, respectively. Following three washes in TBS (0.02 M Tris, 0.2 M NaCl, 0.002 M KCl, pH 7.4) containing 0.025% (*w*/*v*) Triton X-100, sections were blocked with 5% skim milk for 2 h and then incubated with specific primary antibodies: anti-Foxp3 (rabbit IgG, 1:1000; Abcam, Cambridge, UK), anti-IL-17A (rat IgG, 1:100; Thermo Fisher, San Diego, CA, USA) and anti-CD4 (rabbit IgG, 1:100; Abcam; rat IgG, 1:100; BioLegend) for Treg/Th17 staining, anti-CD11c (rabbit IgG, 1:100; Abcam), anti-F4/80 (chicken IgG, 1:100; Abcam), anti-IL-10 (rat IgG, 1:100; Abcam) for macrophage and DC staining, respectively, at 4 °C overnight. Next, sections were washed three times with TBS containing 0.025% Triton X-100 and then incubated with anti-rat (Alexa Fluor 488 conjugated), anti-rabbit (Alexa Fluor 647) and anti-chicken (Alexa Fluor 594) (all three at 1:200; Abcam) secondary anti-IgG polyclonal antibodies. After washing and mounting with antifade mounting medium containing DAPI fluorescent stain (Abcam), sections were visualized and analyzed by the Zeiss LSM 710 Confocal Microscope System (Carl Zeiss, Mainz, Germany) for immunofluorescence. The proportion of IL-10 producing cells, IL-10 producing macrophages (CD11c^+^F4/80^+^IL-10^+^), Th17 cells (CD4^+^IL-17A^+^) and Treg cells (CD4^+^Foxp3^+^) in mice of 4 groups was analyzed using ImageJ software. Excitation and fluorescence wavelengths were selected according to the instruction manual.

### 2.6. RNA Isolation and Quantitative Real-Time PCR

After stimulation with bifidobacterial strains, total RNA was isolated from RAW264.7 macrophage cells using TRIzol reagent (Invitrogen, Carlsbad, CA, USA), and first-strand cDNA was reverse-transcribed at 37 °C for 1 h, 95 °C for 5 min and 4 °C for 5 min using a Promega/GO TaqG2 reagent kit (Pharmacia Biotech, Southampton, UK). Real-time RT-PCR was performed using SYBR Green Master Mix (TaKaRa, Kyoto, Japan). Gene expression of *Tlr1*, *Tlr2* and *Tlr6* was normalized to the expression of the housekeeping gene Gapdh and the results reflect the fold increase relative to the control sample using the ddCT method and GAPDH as the endogenous control. Primers used are listed in Table 2.

### 2.7. Western Blot Analysis

RAW 264.7 macrophages with or without stimulation by bifidobacterial strains were washed twice with PBS and gently scraped off the dish. Total protein was isolated using cell lysis buffer (Biyuntian Biotech) and after centrifugation, the protein in the supernatant was quantified with an enhanced BCA protein assay kit (Biyuntian Biotech). Soluble protein was mixed with 5 × SDS loading buffer (250 mM Tris–HCl pH 6.8, 10% *w*/*v* SDS, 0.5% *w*/*v* bromophenol blue, 50% *v*/*v* glycerol, 5% *w*/*v* β-mercaptoethanol) and separated by 12% SDS–PAGE, and then transferred to a nitrocellulose membrane using a semi-dry transmembrane system. Phosphor-ERK (1/2) and phosphor-p38 MAPK mouse monoclonal antibody (Cell Signaling Technology, Danvers, MA, USA) and GAPDH mouse monoclonal antibody (Biyuntian Biotech) were used to detect the expression of phosphor-ERK (1/2), phosphor-p38 and GAPDH (used as a control), respectively, according to standard western-blot procedures. The pixel intensity was determined using Tanon-1600 GIS analysis software (Tanon, Shanghai, China).

### 2.8. Chromatin Immunoprecipitation (ChIP) Analysis

RAW264.7 cells were stimulated with or without bifidobacterial strains for 3 h and fixed with 1% formaldehyde for 10 min at 37 °C. Following quenching with 125 mM glycine for 5 min, fixed cells were sonicated for 9 min of 0.5 s on/0.5 s off pulses in SDS lysis buffer [50 mM Tris–HCl, pH 8.0, 10 mM EDTA, 1% SDS]. Sonicates were diluted in ChIP dilution buffer and immunoprecipitated with 5 g of anti-acetyl-histone H3 antibody (Millipore, Bellerica, MA, USA) or isotype control overnight at 4 °C. Protein A agarose with salmon sperm DNA was added and rotated for an additional 2 h to capture antibody–protein–DNA complexes. After washing beads with low and high salt, LiCl, and TE buffer (10 mM Tris, 1 mM EDTA, pH 8.0), the protein–DNA complexes were eluted using 1% SDS in 0.1 M NaHCO_3_ buffer and disrupted by heating at 65 °C overnight in 0.2 M NaCl buffer. Recovered DNA was purified with HiBind^®^ DNA Mini Columns (Omega Bio-tek, Norcross, GA, USA) and analyzed by qPCR using specific primers (Table 2).

### 2.9. EPS Isolation and Immune Response Assay

The total bacterial EPS yields of different strains during log-phase growth were detected with concanavalin A–Alexa Fluor 488 (Invitrogen) [31].

EPSs were isolated from bifidobacterial strains using a modification of the procedure described by Hidalgo-Cantabrana and co-workers [32]. Bifidobacterial strains were cultured on the surface of agar–MRS supplemented with 0.05% L-cysteine for 5 days and collected using ultrapure water. Next, the same volume of 2 M NaOH was added to the bacterial suspension and stirred for 16 h at room temperature. After centrifugation, the supernatant containing released EPS was mixed with two volumes of chilled absolute ethanol for 48 h at 4 °C. The precipitated EPS was collected by centrifugation, resuspended in water and dialyzed (in cellulose membrane with a 8–14 kDa molecular weight cutoff; Beijing Science & Technology Co. Ltd., Beijing, China) against ultrapure water for 3 days at 4 °C, then lyophilized to obtain the EPS powder. The EPS (0.2 mg/mL) was used for stimulated RAW264.7 macrophages, or together with bifidobacterial strains for splenocyte coculture (5:1 cell/cell, 1 × 10^7^ cfu:2 × 10^6^ cell) in DMEM or RPMI-1640 culture medium for 72 h, at 37 °C, in 5% CO_2_ atmosphere.

### 2.10. Inhibition of ERK and p38 Phosphorylation

Anti-mouse TLR2 antibody at a concentration of 1 mg/mL (BioLegend) and specific chemical inhibitors for ERK1/2 MAPK (PD98059) and p38 MAPK (SB203580) (Selleck Chemicals LLC, Houston, TX, USA) at a concentration of 10 μM in assays were precultured with RAW264.7 cells for 2 h before stimulation with bifidobacterial strains. Anti-mouse IL-10 antibody (BioLegend) was added to the coculture of bifidobacterial strains and splenocytes.

### 2.11. Statistical Analysis

All biochemical and cell culture assays were performed in at least 3 or 6 independent repeats for each experiment. Statistical analysis of differences between groups was performed using one-way ANOVA. *P* ≤ 0.05 was considered to be statistically significant. Data are presented as means ± SEM of the independent repeats in each experiment.

## 3. Results

### 3.1. B. adolescentis Strains Stimulate Different Cytokine-Secretion Profiles in RAW264.7 Cells

**Figure 1:**
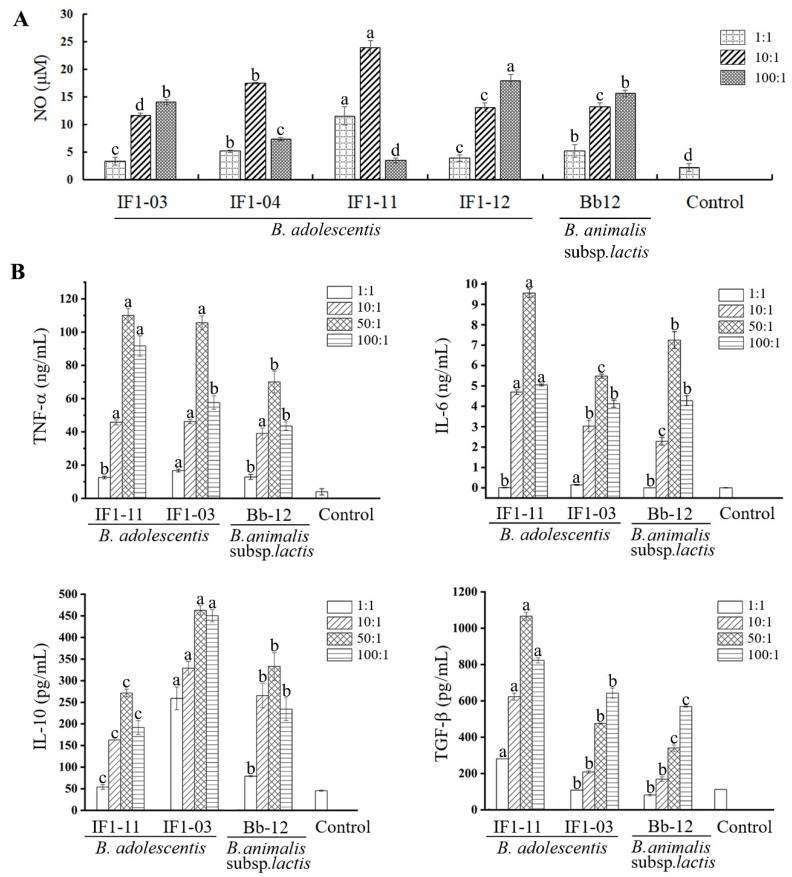
NO and cytokine secretion profiles of RAW264.7 macrophages stimulated by bifidobacterial strains. RAW264.7 macrophages were cocultured with *B. adolescentis* IF1-11, *B. adolescentis* IF1-03 or *B. animalis* subsp. *lactis* Bb12 for 24 h at the indicated ratios. The supernatant NO (μM) was analyzed by the Griess assay (**A**) and cytokines by ELISA (**B**). All strains were prepared at a density of 5 × 10^5^–10^7^ cfu/mL in DMEM, and RAW264.7 macrophage cells were at 5 × 10^5^ cell/mL. Ratios—1:1, 5 × 10^5^ cfu:5 × 10^5^ cell; 10:1, 5 × 10^6^ cfu:5 × 10^5^ cell; 50:1, 2.5 × 10^7^ cfu:5 × 10^5^ cell; 100:1, 5 × 10^7^ cfu:5 × 10^5^ cell, per reaction respectively. Data are shown as mean ± SD for three independent experiments, significant differences in each ratio group are labelled with different letters by one-way ANOVA, *p* < 0.05. TNF-α: Tumor Necrosis factor, IL-6: Interleukin 6, IL-10: Interleukin 10, TGF-β: Transforming growth factor β.

The four *B. adolescentis* strains previously isolated from infant feces with different bacterial phenotypes for aggregation property and intestinal epithelial cell adhesive capacity (Table 1) [30] were assayed for their elicitation of murine macrophage RAW264.7 cell secretion of NO and cytokines when stimulated at different bacterial densities (Figure 1A). NO yields of the macrophages stimulated by *B. adolescentis* IF1-03 and IF1-12 increased with bacterial density (1 to 100:1; 5 × 10^5^–10^7^ cfu:5 × 10^5^ cells); *B. adolescentis* IF1-11 and IF1-04 displayed peak NO induction at a ratio of 10:1 (5 × 10^6^ cfu:5 × 10^5^ cell) (Figure 1A). Strain IF1-11 stimulation resulted in a 1.5–2 times higher NO yield compared to the other strains, whereas IF1-03 showed the lowest NO-eliciting ability for RAW264.7 cells. 

Macrophages secrete cytokines, including TNF-α, IL-6, TGF-β and IL-10, which play a key role in initiating and restraining inflammation, thereby stimulating the adaptive immune response and CD4^+^ T-cell differentiation, which may influence the severity of IBD. As NO stimulation diverged for strains IF1-03 and IF1-11, as did the aggregation property of these two strains, which might indicate the different surface components on them, the cytokine patterns of the RAW264.7 cells were further investigated. As shown in Figure 1B, *B. adolescentis* IF1-03 activation of RAW264.7 cells resulted in a similar cytokine secretion profile to *B. animals* Bb-12 for TNF-α and IL-6 at most bacterial cell density to macrophage cell ratios, except at the ratio of 50:1. There were much higher amounts of IL-10 and a mildly higher, but significant, amount of TGF-β secreted by RAW264.7 stimulated with *B. adolescentis* IF1-03 compared to *B. animals* Bb-12. While *B. adolescentis* IF1-11 stimulated RAW264.7 to secrete a much higher amount of IL-6 and TGFβ compared with *B. adolescentis* IF1-03, and a similar amount of TNF-α at bacterial cell density to macrophage cell ratios of 10–50:1. Therefore, the results indicate that the two *B. adolescentis* strains elicited different IL-6, IL-10 and TGF-β patterns, which are critical cytokines for Treg/Th17 cell homeostasis. We hypothesized that the two *B. adolescentis* strains might have different effects on CD4^+^ T cell differentiation to Treg/Th17 cells in tissue-resident macrophages mediating the adaptive immune response.

### 3.2. Induction of Treg/Th17 Cell Lineages from Splenocytes by B. adolescentis Strains

**Figure 2:**
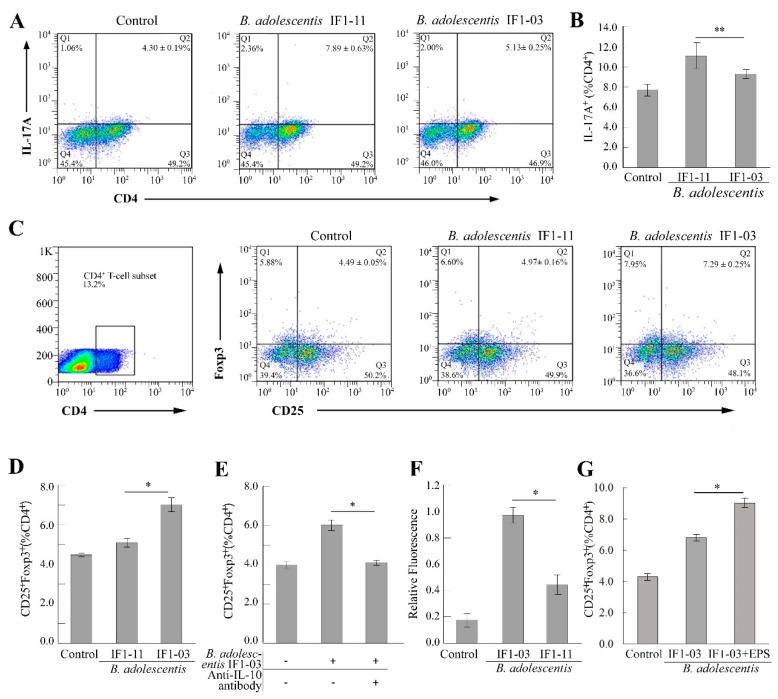
Effect of two *B. adolescentis* strains on differentiation and proliferation of Th17/Treg cells in coculture with splenocytes. Splenocytes isolated from BALB/c mice were stimulated with *B. adolescentis* IF1-11, *B. adolescentis* IF1-03 or left untreated (control) for 72 h. (**A**) Representative flow cytometric dot plot and (**B**) proportion of Th17 cells (CD4^+^IL-17A^+^) in spleen lymphocytes. (**C**) Representative flow cytometric dot plot and proportion of Treg cells (CD4+CD25+Foxp3+) to CD4+ T cells. SSC-H (side scatter-height) the basic parameter indicating cell internal complexity, FL2-H (Foxp3, PE), FL3-H (CD4, PerCp—Cy5.5), and FL4-H (CD25, APC) emitted fluorescence signal height of different fluorescent dyes, by flow cytometry analysis. (**D**) Proportion of Treg cells (CD4^+^CD25^+^Foxp3^+^) in spleen lymphocytes stimulated with strain IF1-11 or IF1-03. (**E**) Effect of the addition of anti-IL-10 antibody on the induction of Treg cells induced by *B. adolescentis* IF1-03. (**F**) Amount of EPSs in *B. adolescentis* IF1-03 and *B. adolescentis* IF1-11. (**G**) Proportion of Treg cells (CD4^+^CD25^+^Foxp3^+^) in splenocytes stimulated with *B. adolescentis* IF1-03 cells alone or in the presence of IF1-03 EPS for 72 h. Data are shown as mean ± SD for five independent experiments and the significances between two *B. adolescentis* strains (**A**–**F**), with or without the addition of *B. adolescentis* IF1-03 EPS (**G**) were analyzed by one-way ANOVA, (* *p* < 0.05, ** *p* < 0.01).

Regulation of the polarization and expansion of effector and regulatory T cells is a crucial response initiated by the innate immune system through antigen presentation and secretion of cytokines by antigen-presenting cells, such as DCs and macrophages, when interacting with intestinal microbes. To assess the bifidobacterial strains’ immunomodulatory capacity on T cells, splenocytes (2 × 10^6^ cell/mL) isolated from 6- to 8-week-old BALB/c mice were stimulated with *B. adolescentis* IF1-03 or IF1-11 cells at a bacterial cell density to splenocyte ratio of 5:1, combined with anti-CD3/CD28 antibodies. T cell activation, and the expansion of Th17 and Treg cells, were analyzed by flow cytometry. As shown in Figure 2, splenocyte coculturing with or without *B. adolescentis* IF1-03 or IF1-11 resulted in a different proportion of Treg/Th17 cells to the total number of CD4^+^ T cells (Figure 2A–D). The IF1-11 strain induced a significant increase in the proportion of Th17 cells (CD4^+^IL-17A^+^) (Figure 2A,B), whereas IF1-03 induced a significant increase in the proportion of Treg cells (CD4^+^CD25^+^Foxp3^+^) to CD4^+^ T cells (Figure 2C,D). The increase in Treg cells in spleen lymphocytes might correspond to the large amount of IL-10 secreted by the macrophages stimulated with *B. adolescentis* IF1-03. Therefore, we used anti-mouse IL-10 antibody to block the IL-10, and the proportion of Treg cells to CD4^+^ T cells decreased to the basal level of control splenic lymphocytes (Figure 2E). The two *B. adolescentis* strains showed differential cell adhesion and auto/coaggregation (Table 1), which was strongly related to the EPS on the cell wall. The fluorescent EPS assay on growing bifidobacteria showed that live *B. adolescentis* IF1-03 cells produce more EPS than IF1-11 cells (Figure 2F). This suggested that EPS produced by *B. adolescentis* IF1-03 contains abundant α-mannopyranosyl and α-glucopyranosyl residues, which are selectively bound by the lectin-conjugated fluorescent probe conjugate concanavalin A–Alexa Fluor 488 [30]. With the addition of EPS, the proportion of Treg cells (CD4^+^CD25^+^Foxp3^+^) induced from BALB/c mouse splenocytes stimulated by *B. adolescentis* IF1-03 increased further (Figure 2G), indicating that the EPSs of the probiotic strain play a main or superposed role in the innate and adaptive immune response, and can be recognized by the macrophages or DCs as P/MAMPs in the probiotic–symbiont immune system.

### 3.3. Effect of B. adolescentis Strains on Mouse DSS-Colitis Protection

**Figure 3:**
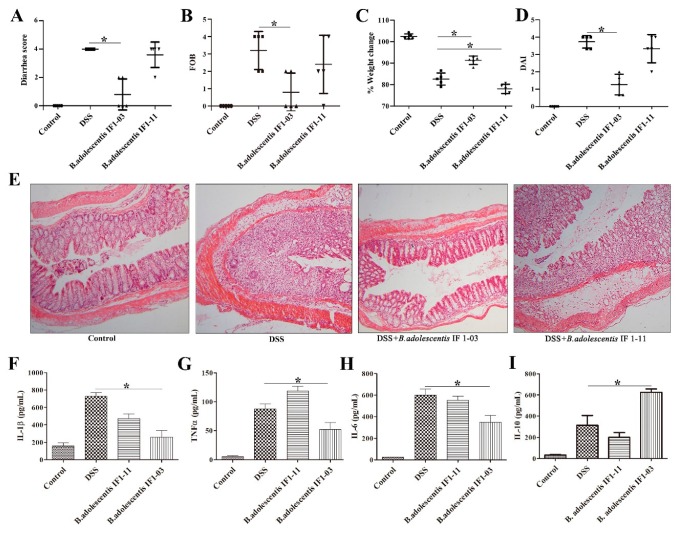
Effect of two bifidobacterial strains on DSS-colitis C57BL/6 mice. (**A**) Diarrhea, (**B**) bloody feces, tested by fecal occult blood (FOB) test kit, (**C**) weight variation between day 9 (oral DSS administration) and day 16 (death) and (**D**) disease activity index (DAI) of the different mouse treatment groups. Dot plots are representative of 5 individual mice in each mouse group. (**E**) Representative histological appearance of colonic tissues of C57BL/6 mice in the different treatment groups. Histochemical micrograph was taken at 10 × 10 magnification. (**F**) IL-1β, (**G**) TNF-α, (**H**) IL-6 and (**I**) IL-10 secretion by colonic lamina propria cells of different mouse groups. C57BL/6 mouse treatment groups: Control, oral administration of skim milk only for 15 days; DSS, DSS-colitis model group, 6 days DSS ad libitum started on day 9; *B. adolescentis* IF1-11 and IF1-03 groups, oral administration of strain IF1-11 or IF1-03 for 15 days, plus 6 days DSS ad libitum started on day 9 for protection against DSS-colitis, respectively. Data are shown as mean ± SD for three independent experiments, significant difference between indicated groups means by one-way ANOVA (* *p* < 0.05). IL-1β: Interleukin 1β, TNF-α: Tumor Necrosis factor, IL-6: Interleukin 6, IL-10: Interleukin 10.

To investigate whether the differential induction of Treg/Th17 cells by *B. adolescentis* strains IF1-03 and IF1-11 provides substantial protection against DSS-induced colitis by regulating anti-inflammatory and immunoregulatory effects in vivo, the murine DSS-colitis model was used to address the probiotics’ influence on IBD. The main clinical indexes of DSS-colitis—diarrhea, bloody feces, weight loss and disease activity index—of the different mouse groups are shown in Figure 3. At the end of the experiment, mice of the DSS-colitis model group and the *B. adolescentis* IF1-11 protection group both lost around 17% to 21% of their body weight and developed severe bloody diarrhea, compared to the control group (Figure 3A–D). In contrast, mice of the *B. adolescentis* IF1-03 protection group lost around 8% of their body weight and only 2 of the 5 mice developed slight diarrhea, indicating significant protection against DSS-induced colitis symptoms (Figure 3A–D). Histopathology revealed that the microscopic structure of the colon villi of the *B. adolescentis* IF1-03 protection group mice was much improved over that of the DSS-colitis model and IF1-11 strain protection mice. *B. adolescentis* IF1-03 reduced the area of ulceration and thickening of the intestinal wall compared to the IF1-11 strain protection and DSS-colitis model mice (Figure 3E). The cytokines produced by the colonic lamina propria cells of the different mouse groups were measured. DSS-colitis model mice produced high levels of the proinflammatory cytokines IL-1β, IL-6 and TNF-α, and *B. adolescentis* IF1-03 mice produced lower IL-1β, IL-6 and TNF-α, and elevated levels of the anti-inflammatory IL-10, compared to the DSS-colitis model and *B. adolescentis* IF1-11 protection mice (Figure 3F–I). *B. adolescentis* IF1-11 showed weak protection ability for proinflammatory factor increments, with increased TNF-α and decreased IL-10 secretion by the colonic lamina propria compared to the DSS-colitis model (Figure 3F–I). 

**Figure 4:**
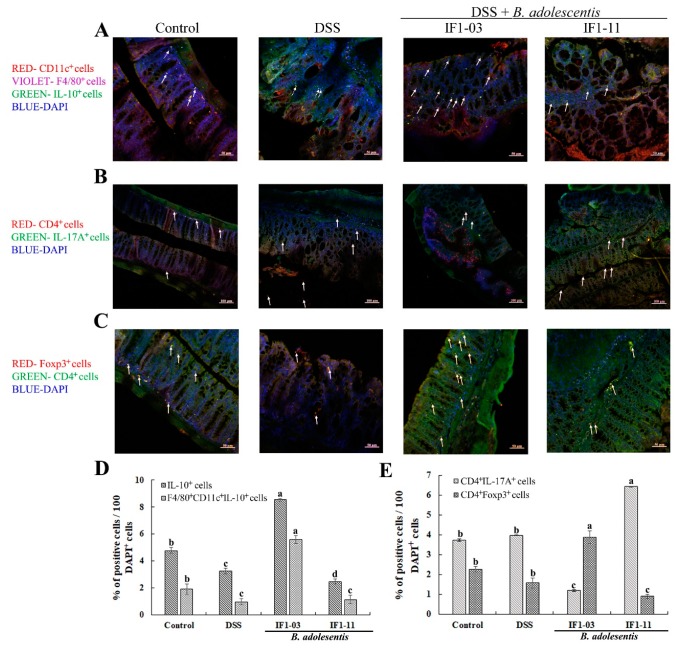
Effect on induction and distribution of Treg (CD4^+^Foxp3^+^)/Th17 (CD4^+^IL17A^+^) cells and IL-10-producing CD11c^+^ DCs or F4/80^+^ macrophages in colon tissue of C57BL/6 control mice (placebo, per os), DSS-colitis model mice (6 days DSS, ad libitum, days 9–15) or *B. adolescentis* strain IF1-03/IF1-11 protection of DSS-colitis mice (15 days bifidobacterial cells per os, with DSS ad libitum on days 9–15). Mouse colon sections were stained with CD11c (red dots), IL-10 (green dots) and F4/80 (violet dots) antibodies (**A**), CD4 (red dots) and IL-17A (green dots) antibodies (**B**), or CD4 (green dots) and Foxp3 (red dots) antibodies (**C**). Microfluorescence image visualized by Zeiss LSM 710 Confocal System. In (**A**), representative DCs (CD11c^+^F4/80^−^IL-10^+^) (dotted arrow) and macrophages (CD11c^+^F4/80^+^IL-10^+^) (solid arrow) in the four mouse groups are indicated. In (**B**,**C**), representative Th17 (CD4^+^IL17A^+^) and Treg (CD4^+^Foxp3^+^) cells in the four mouse groups are indicated by solid arrows. Bars = 50 μm (200×) in (**A**,**C**) and 100 μm (100×) in (**B**), respectively. Proportion of IL-10 producing cells (IL-10^+^), IL-10 producing macrophages (CD11c^+^F4/80^+^IL-10^+^), Th17 cells (CD4^+^IL-17A^+^) and Treg cells (CD4^+^Foxp3^+^) in mice of the four groups. IL-10 producing cells, IL-10 producing macrophages (CD11c^+^F4/80^+^IL-10^+^) (**D**), Th17 cells (CD4^+^IL-17A^+^) and Treg cells (CD4^+^Foxp3^+^) (**E**) in mice of the four groups was numbered and analyzed by using ImageJ software. Data are shown as mean ± SD for three independent experiments and those with same signs and labelled with different letters are significantly different by one-way ANOVA (* *p* < 0.05).

Immunofluorescence microscopy and histochemical assays showed an abundance of macrophages accumulated in the colon of DSS-colitis mice, and a higher number of IL-10-producing cells appeared in the *B. adolescentis* IF1-03 protection mouse colon sections (Figure 4A,D). As shown in Figure 4A, where the IL-10-producing macrophages (CD11c^+^F4/80^+^IL10^+^) and DCs (CD11c^+^F4/80^−^IL10^+^) are signalled by solid and dotted arrows, respectively, the IL-10-producing macrophages were significantly induced in *B. adolescentis* IF1-03 protection group mice (Figure 4A,D). More Th17 cells (CD4^+^IL17A^+^) were distributed in the colonic villi of the *B. adolescentis* IF1-11 protection groups compared to control mice and the *B. adolescentis* IF1-03 protection group (Figure 4B,E). Treg cells (CD4^+^Foxp3^+^) were observed in higher numbers in the colonic villi of the *B. adolescentis* IF1-03 protection group than in the three other mouse groups (Figure 4C,E). 

Taken together, *B. adolescentis* IF1-03, rich in strain-specific EPSs, manifested an anti-inflammatory immunoregulatory effect by skewing the adaptive immune response toward CD4^+^ T cell expansion to Treg cells, and stimulated low NO and IL-6 secretion and high IL-10 secretion by macrophages, compared to *B. adolescentis* IF1-11, both in vitro and in vivo.

### 3.4. Activation of TLR2 and MAPK-Signaling Cascade by the Two B. adolescentis Strains

**Figure 5:**
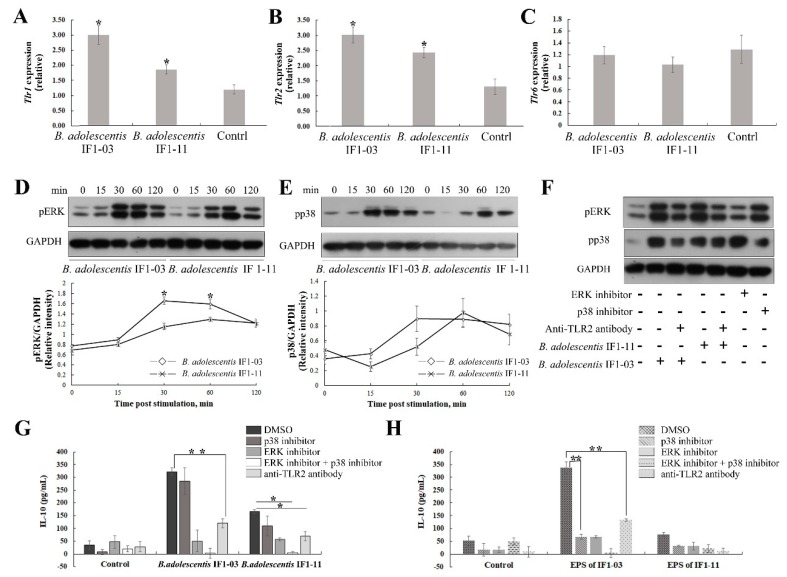
Activation of TLR2 and subsequent ERK/p38- MAPK signaling in RAW264.7 macrophages after stimulation with two bifidobacterial strains. Total RNA of macrophages stimulated with *B. adolescentis* IF1-11 and *B. adolescentis* IF1-03, and control macrophages, was isolated at 4 h for cDNA synthesis and assay. Expression of *Tlr1* (**A**), *Tlr2* (**B**) and *Tlr6* (**C**) was analyzed by qRT-PCR. Western blot analysis of the phosphorylated ERK (pERK) (**D**) and phosphorylated p38 (pp38) (**E**) in treated macrophages at the indicated times. Western blot analysis of pERK and pp38 of *B. adolescentis* IF1-03 and IF1-11-stimulated macrophages in the presence of anti-mouse TLR2 antibody, and ERK/p38-MAPK signal pathway inhibitors (**F**). IL-10 synthesis and secretion in macrophages stimulated with *B. adolescentis* IF1-11 and IF1-03 strains, in the presence of anti-mouse TLR2 antibody and specific chemical inhibitors of ERK (PD98059, 10 μM) and p38 (SB203580, 10 μM) phosphorylation (**G**). EPSs of different strains stimulate IL-10 production of macrophages in the presence of TLR2 antibody and inhibitors, respectively. IL-10 concentration in supernatants of cell cultures was determined by ELISA (**H**). Data are shown as mean ± SD for three independent experiments, statistically significant difference is indicated (* *p* < 0.05, ** *p* < 0.01).

The *B. adolescentis* strain-dependent specificity for P/MAMP recognition, signal cascade for macrophage maturation and polarization and inflammatory immunoregulatory cytokine induction were investigated. We compared the transcriptional regulation of *Tlr2*, *Tlr1* and *Tlr6* in RAW264.7 cells stimulated by the two *B. adolescentis* strains. Both IF1-03 and IF1-11 strains successfully upregulated *Tlr1* and *Tlr2* expression in RAW264.7 cells (Figure 5A,B). While IF1-11 stimulated higher *Tlr2* (2.4-fold) than *Tlr1* (1.8-fold), and the IF-03 strain upregulated *Tlr1* and *Tlr2* expression equally (3-fold), and more strongly than strain IF1-11. *Tlr6* expression was not affected (Figure 5C). Stimulation of macrophage RAW264.7 cells by *B. adolescentis* IF1-03 induced stronger phosphorylation of ERK and a faster rate of p38 phosphorylation in RAW264.7 cells than strain IF1-11 (Figure 5D,E). TLR2 blockage, and ERK and p38 phosphorylation inhibitors affected pERK and phosphorylated p38 abundance levels in *B. adolescentis*-treated macrophages (Figure 5F). As TLRs are the main PRRs recognizing extracellular signals, TLR2–ERK/p38 MAPK signaling for IL-10 synthesis was further confirmed by blocking with anti-TLR2 antibody and using specific chemical inhibitors of the ERK/p38 MAPK pathways (Figure 5G,H) in RAW264.7 cells. Induction of macrophages for IL-10 production by *B. adolescentis* IF1-03 and its EPSs was highly dependent on TLR2 activation and phosphorylation of ERK and p38. Bifidobacterial cell-induced macrophages and their IL-10 production was more inclined to the TLR2–ERK signal pathway; p38 MAPK inhibition only slightly inhibited final IL-10 production, and only double inhibition of the ERK/p38 MAPK pathway resulted in complete blockage of IL-10 secretion (Figure 5G). In the EPS-treated macrophages, IL-10 yield was substantially decreased by p38 MAPK inhibition, and the sensitivity to p38 inhibitor was strongly enhanced, equaling the extent of ERK inhibition (Figure 5H). The results indicated that macrophage maturation, polarization and IL-10 production are driven by the *B. adolescentis* IF1-03 cells, and the two branches of the TLR2/1–ERK/p38 MAPK signaling pathway are balanced by strain-specific EPSs, which may be complexed with other P/MAMPs.

### 3.5. Induction of Transcriptional Regulatory Element in Il10 Gene Locus

**Figure 6:**
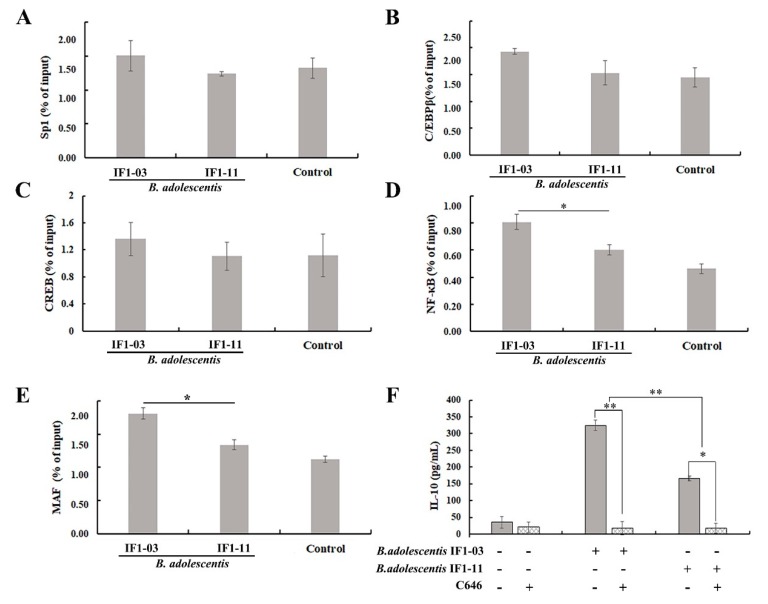
*B. adolescentis* strain-stimulated RAW264.7 macrophages show active chromosome remodeling related to targeted gene promoter combination with transcriptional factors NF-κB and others, and IL-10 production. Macrophages were stimulated with *B. adolescentis* IF1-11, *B. adolescentis* IF1-03 or left untreated for 3 h (control) and the level of H3K9 acetylation on sites of transcription factor–genomic DNA interaction sequence at the *Il10* proximal promoter locus was determined by qPCR with specific primers (Table 1). (**A**) Sp1, (**B**) C/EBPβ, (**C**) CREB, (**D**) NF-κB, (**E**) MAF, and (**F**) IL-10 yield in macrophages treated with *B. adolescentis* strains, and inhibition of H3K9 acetylation with C646. IL-10 was assayed by ELISA. Data are shown as mean ± SD for three independent experiments (* *p* < 0.05, ** *p* < 0.01). Sp1: Specific protein 1, C/EBPβ: CCAAT/enhancer binding protein β, CREB: c-AMP responsive element binding protein, NF-κB: Nuclear factor κB.

To address the accessibility of transcription factors at the *Il10* proximal promoter locus of macrophage cells, ChIP assays were conducted with antibody against acetylated histone H3K9 to reveal differences between the two strains of *B. adolescentis* in the last step toward turning on the targeted *Il10* via the TLR2–ERK/p38 MAPK/NF-κB signaling axis. Several binding sites for transcriptional regulators, such as NF-κB, MAF, CCAAT/enhancer binding protein β (C/EBP), cAMP responsive element-binding protein (CREB) and specific protein (SP1 and SP3) were investigated. As shown in Figure 6, upon *B. adolescentis* IF1-03 stimulation of RAW264.7 macrophages, H3 on the *Il10* promoter was acetylated on more transcription factor-binding sites compared to *B. adolescentis* IF1-11 stimulation. In addition, there were significant differences in acetylation on the binding sites of MAF and NF-κB between stimulation by *B. adolescentis* IF1-03 and IF1-11 (Figure 6D,E), while no significant different acetylation level on the binding sites of C/EBP, CREB, SP1 and SP3 was detected (Figure 6A–C). Inhibition of H3K9 acetylation with histone acetyltransferase inhibitor C646 effectively reduced the IL-10 yield of RAW264.7 macrophages stimulated by *B. adolescentis* IF1-03 (Figure 6F).

## 4. Discussion

The intestinal microbiome works as a signaling hub that can integrate environmental input from diet and xenobiotics with genetic and immune signals to affect the host intestinal mucosal and system responses [15]. Since the successful application of fecal microbiota transplantation to ameliorate specific disease states, probiotic research has turned to developing probiotics that address specific consumer needs and issues, providing, for instance, specific immunomodulatory effects. Numerous studies have proven the amelioration of chronic inflammation and some metabolic syndromes through immunoregulation by bacterial strains; however, scientific and rational selection criteria are essential for the application of new strains because they diverge somewhat in different situations [33]. The Treg/Th17 axis has distinct effects on the establishment of system tolerance to a large spectrum of environmental antigens [11]. The genus *Bifidobacterium* is one of the most well studied and widely applied probiotic bacteria, especially in the modulation of gut and system immune responses. A recent study showed an association between high adhesion to epithelial cells and Th17 cell induction, and a subsequent study identified *B. adolescentis* L2-32 as the first human-source commensal inducing Th17 cells, analogous to segmented filamentous bacteria in rodents [12,17]. Therefore, investigation of the immunoregulatory properties and molecular and cellular mechanisms that are critical for the specific function of these strains are of great importance [13,15].

In this study, we investigated the immunoregulatory effect on the Treg/Th17 axis of four *B. adolescentis* strains isolated from infant feces that presented distinct adhesive abilities among 13 strains in in-vitro experiments [30]. Following stimulation with these strains, RAW264.7 cells showed differential secretion of cytokines, mainly IL-10, IL-6 and TGF-β, which are critical to the induction of Treg and Th17 cell differentiation [19,20,21,34]. The effect on the polarization and proliferation of different CD4^+^ T-cell subsets in splenocytes stimulated with *B. adolescentis* IF1-03 and IF1-11 was manifested as a variation in the proportions of Treg and Th17 cells, consistent with the function of the differentially secreted cytokines by RAW264.7 macrophages. IL-6 has been reported to induce activation of the Stat3/MAPK pathway and the subsequent expression of target genes, steering the TLR/MYD88/ MAPK/NF-κB axis toward regulation of the Treg/Th17 balance [34,35,36]. *B. adolescentis* IF1-03 stimulated macrophage maturation to produce higher levels of IL-10 and lower levels of IL-6 and TGF-β, consistent with the upregulation of Treg cells in DSS-colitis mice in vivo, and splenocytes in vitro. *B. adolescentis* IF1-11, on the other hand, stimulated macrophages to secrete higher levels of IL-6 and TGF-β and lower levels of IL-10, skewing the CD4^+^ T cells to Th17 cells. The latter enhance clearance of extracellular pathogens and are induced by colitogenic bacteria. Th17 cells have a dual role in human health: they cause increased permeability and bacterial infection in mice devoid of IL-17 signaling and there is an elevated frequency of Th17 cells in IBD and other extraintestinal autoimmune disorders [37]. Adhesive *B. adolescentis* L2-32 exacerbates autoimmune arthritis due to its induction of Th17 cells [17]. In the present study, administration of *B. adolescentis* IF1-03 increased the distribution of Treg cells, and IL-10 producing macrophages in the lamina propria, protecting the mice from DSS-induced colitis in vivo, whereas *B. adolescentis* IF1-11 upregulated the differentiation of IL-17A without any amelioration of DSS-colitis, in accordance with the in vitro results and physiological functions of Treg and Th17 cells [38]. Taken together, these two *B. adolescentis* strains with similar adhesion to epithelial cells but different auto-aggregation properties and EPS yields, differentially induced Treg/Th17 cells; this differs somewhat from the association between adhesion and Th17 cell induction proposed in previous reports [11,16].

IL-10 is responsible for maintaining the expression and function of Foxp3 in Treg cells [21]. IL-10 secreted by lamina propria macrophages has been shown to be important for maintaining Foxp3 expression in a mouse model of colitis [39]. In our study, we observed upregulation in the number of macrophages secreting IL-10 within the colonic tissue of mice stimulated with *B. adolescentis* IF1-03. The regulation of IL-10 production is mainly through the activation of signaling cascades, with the TLR2/1-ERK/p38 MAPK/NF-κB cascade dominating in macrophages, and is induced through chromatin remodeling of the targeted *Il10* promotor region [40]. In cells deficient in tumor progressing locus 2, which is an upstream activator of ERK and regulated by the NF-κB family member p105, and in the presence of a chemical inhibitor of ERK or in ERK-deficient cells, IL-10 production is limited [41]. Moreover, p38 has been shown to induce the activation of mitogen- and stress-activated kinases (MSKs), which are involved in feedback regulation of p38 by the phosphatase Dusp1 in murine cells treated with LPS [42]. Furthermore, ERK and p38 may cooperate to regulate the production of IL-10 in TLR4-stimulated macrophages by activating MSK1 and MSK2 [42]. In our study, the induction of IL-10 by *B. adolescentis* IF1-03 was dependent on the activation of ERK/p38 MAPK signaling; co-inhibition of ERK and p38 signaling blocked the expression of *Il10*. In macrophages, TLRs are the main PRRs recognizing a range of ligands post-stimulation, such as PAMPs, MAMPs and others [25,43]. Among the TLRs, the ligation of TLR2, 4, 5, 7 and 9 has been shown to induce the production of IL-10 in human and murine macrophages [24,40]. In our study, we determined TLR2 as the core responding receptor in recognizing the two bifidobacterial strains by investigating the expression changes of *Tlr1* and *Tlr2* in RAW264.7 macrophages stimulated by *B. adolescentis* IF1-03, with downstream TLR ligation, and ERK/p38 MAPK positively regulating IL-10 production in macrophages. We checked the accessibility of transcription factors at the *Il10* proximal promoter locus and found that the binding sites of MAF and NF-κB were significantly acetylated in RAW264.7 macrophages stimulated with *B. adolescentis* IF1-03 compared to those stimulated with *B. adolescentis* IF1-11. These results suggest that the mechanism by which *B. adolescentis* IF1-03 induces mouse macrophage *Il10* expression also includes strengthening the chromosome-remodeling modification of histone acetylation of the H3K9 region to make the *Il10* promoter more accessible to transcriptional regulators.

MAMPs on bacteria are recognized by PRRs when immune cells are stimulated [44]. As EPSs are critical for bacterial aggregation [45] and immunoregulation [46], we investigated the immunoregulatory response of the crude EPS isolated from these two strains. Consistent with the effect displayed by the strains, the addition of EPSs in a coculture of *B. adolescentis* IF1-03 and splenocytes increased the proportion of Treg cells and stimulated IL-10 production by the macrophages. In recent years, the immunoregulatory response of bifidobacterial EPSs has been deciphered, and high-molecular-weight EPS and rhamnose contents were shown to induce the anti-inflammatory cytokine profile [46]. Moreover, *B. animalis* subsp. *lactis* IPLA-R1, a mutated strain of *B. animalis* subsp. *lactis* A1 that expresses high-molecular-weight EPSs, was proven to induce a significant increase in suppressor-regulatory TGF-β and a reduction in proinflammatory IL-6 [47]. Separation of the crude EPSs of the two *B. adolescentis* strains by Sephadex CL-6B gel filtration chromatography showed that strain IF1-03 has a much higher abundance of high-molecular-weight EPS fractions than *B. adolescentis* IF1-11 (data not shown), which might be the key component of strain-specific P/MAMPs, or sugar-complexed PAMPs. In the *B. adolescentis* EPS-stimulated macrophages, IL-10 yield was elevated, whereas p38 phosphorylation blockage greatly decreased this yield, indicating that TLR2 recognition activated downstream signaling of the TLR2–ERK MAPK/NF-κB axis and cross-talk with the TLR2–p38 MAPK/NF-κB axis [24,42,48] was strengthened. Although live bacteria probably do not pass the mesenteric lymph node filter [49], gut microbiota-derived peptidoglycan can be detected in the serum of mice, indicating that microbial ligands of PRRs circulate throughout the body at sufficient concentrations to influence the function of immune cells at non-mucosal sites [50].

## 5. Conclusions

Taken together, our findings suggest that probiotic *B. aldolescentis* displays a strain-dependent immunoregulatory effect, which is tightly associated with the strain’s specific sugar-complexed P/MAMPs. The TRL2–ERK/MAPK/NF-κB signaling pathway, which is specifically involved in innate immune cell maturation and polarization, transduces the probiotics’ adaptive immunoregulation to the Treg/Th17 axis. The *B. aldolescentis* IF1-03 strain with high-molecular-weight EPSs, high epithelial cell adhesion and low auto-aggregation stimulated macrophage polarization toward immunosuppressive-type macrophages with anti-inflammatory cytokine IL-10 secretion and skewing of naïve T cells toward Treg cells in colonic lamina propria, in vivo, and splenocytes, in vitro. As an increasing variety of disease states and disorders are being found to correlate with the variability of host microbiota, large-scale sequencing projects will further advance our understanding of the link between health and microbiota. *Bifidobacterium* is one example of these variable species, shifting in allergies and IBD. Our study of immune phenotype associations with strain properties might assist in the application of bifidobacterial strains in immune therapies. 

## Figures and Tables

**Table 1:** *Bifidobacterium* strains used in this study.

Strain	Cell Adhesion	Aggregation		Origin
		Auto-Aggregation	Co-Aggregation	
*Bifidobacterium adolescentis*	++	++	+	This lab, Zuo et al., 2015 [30]
IF1-12				Breast-fed infant feces
*B. adolescentis*	+++	+++++	++++	This lab, Zuo et al., 2015 [30]
IF1-11				Breast-fed infant feces
*B. adolescentis* IF1-03	++++	+	++	This lab, Zuo et al., 2015 [30]
				Breast-fed infant feces
*B. adolescentis* IF1-04	++++	+	+	This lab, Zuo et al., 2015 [30]
				Breast-fed infant feces
*B. animalis* subsp. *lactis* Bb-12	++++	+	+	Chr. Hansen (Beijing) Trading Co. Ltd.

**Table 2:** PCR primers used for qPCR.

Gene	Primer Pair Sequence (5′→3′)
C/EBPβ	GAGTGGAGGAAACAATTATTTCTCAATC
	CTGAGGCAGACAGCTGTTCTATGTACA
MAF	GAATCCACAGATGAGGGCCTCTGTAC
	CGCTAAAGAACTGGTCGGAATGAAC
SP1	GAGAGGTAGCCCATACTAAAAATAGCTG
	GTTTTTGTTATTCAGGCTCCTCCTC
CREB	GTAATGCAGAAGTTCATTCCGACCAG
	TTTTATACTGAAGGCTCAGTGGGGC
NF-κB	GAGGAGGAGCCTGAATAACAAAAACC
	AGCAGTGCTGAGCCAGGCATG
TLR1	GTTGTCACTGATGTCTTCAGC
	GCTGTACCTTAGAGAATTCTG
TLR2	CAGCTTAAAGGGCGGGTCAGAG
	TGGAGACGCCAGCTCTGGCTCA
TLR6	CAACTTAACGATAACTGAGAG
	CCAGAGAGGACATATTCTTAG
GAPDH	GCAGTGGCAAAGTGGAGATT
	GTCTTCTGGGTGGCAGTGAT
